# The Effect of Providing Free Eyeglasses on Children’s Mental Health Outcomes in China: A Cluster-Randomized Controlled Trial

**DOI:** 10.3390/ijerph15122749

**Published:** 2018-12-05

**Authors:** Hongyu Guan, Huan Wang, Kang Du, Jin Zhao, Matthew Boswell, Yaojiang Shi, Yiwei Qian

**Affiliations:** 1 Center for Experimental Economics in Education, Shaanxi Normal University, Xi’an 710127, China; hongyuguan0621@gmail.com (H.G.); dukangceee@163.com (K.D.); zhaojinceee@163.com (J.Z.); shiyaojiang7@gmail.com (Y.S.); 2 Freeman Spogli Institute for International Studies, Stanford University, Stanford, CA 94305, USA; kefka@stanford.edu; 3 Department of Economics, University of Southern California, Los Angeles, CA 90089, USA

**Keywords:** myopia, eyeglasses, randomized controlled trial, health technology adoption, mental health

## Abstract

If children with common vision problems receive and use eyeglasses, their educational performance rises. Without proper treatment, visually impaired children may not achieve educational gains and could suffer from poor mental health. We use a randomized controlled trial to study the impact of an eyeglasses promotion program in rural China on the mental health of myopic primary school students. Three measures of mental health are used: learning anxiety, physical anxiety, and scores on the Mental Health Test (MHT). Our empirical analysis showed that on average, the treatment has small and insignificant for learning anxiety and MHT, and a small but significant reduction in physical anxiety. However, subgroup analysis reveals that myopic students who study more intensively see their learning anxiety and physical anxiety reduced after being provided with eyeglasses. In contrast, students with the lower study intensity suffer a rise in learning anxiety after receiving eyeglasses. A potential mechanism for the differing impacts is the increase in teasing reported among low study-intensity students that does not occur for high study-intensity students. Care should be taken to maximize the benefits and minimize the costs of in-school vision programs.

## 1. Introduction 

Nearly half of the disabilities that affect children in developing countries are due to visual impairment [1]. The most common and easily treated cause of visual impairment is uncorrected refractive error. Among all children in the world who are visually impaired by refractive error, approximately half live in China [2]. Although eyeglasses offer a safe, effective, and inexpensive solution to visual impairment caused by refractive error, recent studies have shown that as few as one in six children in lower- and middle-income areas of the developing world who need eyeglasses actually own them [1]. 

In view of the large global burden of uncorrected pediatric refractive error, there is interest in addressing this condition and the subsequent impact on children’s lives. A recent, large randomized controlled trial in rural western China demonstrated that providing eyeglasses could significantly improve children’s educational outcomes [1,3]. The magnitude of the impact has been shown to be comparable to and potentially exceeding other in-school health care interventions, such as de-worming or nutritional supplements [4]. Providing eyeglasses also has been shown to reverse decrements in self-reported visual function in children with even modest abnormalities in visual acuity (<6/9) [5].

Although previous studies have focused mainly on the relationship between the correction of refractive error and educational outcomes, there is also concern that visual impairment and the absence of quality vision care for students may be a source of mental health problems [6]. In particular, the literature highlights competitive education systems as putting adolescents at risk of various mental health problems that may be exacerbated by anything that inhibits learning. For myopic students, the inability to see clearly could be a serious barrier to learning, especially in settings in which the blackboard, a common teaching tool, is positioned at the front of the classroom [1]. The inability to perform may itself be a source of anxiety, creating a cycle of poor achievement and worsening mental health [7]. 

There is a growing literature on the role of vision correction as an educational input [1,3,8]. A number of randomized studies have shown that correcting vision can improve scores on standardized tests. One study, for example, has shown that the provision of glasses in schools boosts standardized test scores among primary school students [1]. A similarly study found that the existence of systematic screening and referrals for free refraction and prescription eyeglasses at a local vision clinic raised test scores for children in surrounding schools [9]. Studies in other settings have shown similar positive results [1,3,4]

There is also a consensus on the link between poor vision and mental health. Studies among adults and the elderly have found that vision correction is associated with better mental health, less worry, reduced frustration, and a higher sense of well-being [10,11]. A smaller literature documents a correlation between uncorrected myopia and mental health among children [12].

However, comparatively little is known about how correcting vision may affect mental health in an educational context. The issue is especially important given the high degree of pressure to perform in many schooling environments, such as China, where only top-performing students in high-stake tests may enroll into high school and university, get recruited for satisfactory jobs, and enjoy opportunities that are off-limits to lower scoring students [13]. The literature shows that competitive education systems put adolescents at risk of various mental health problems that may be exacerbated by anything that inhibits learning. For myopic students, the inability to see clearly could be a serious barrier to learning, especially in settings in which the blackboard, a common teaching tool, is positioned at the front of the classroom [14]. The inability to perform in school might be a source of anxiety, creating a cycle of poor achievement and worsening mental health.

Despite the potential benefits of vision correction for mental health in these settings, there is also the possibility of negative impacts of wearing eyeglasses. Research suggests that a change in personal appearance due to eyeglasses wear can invite teasing from peers [15,16]. A recent study among students in the UK, for example, showed that students with glasses were far more likely to fall victim to bullying and name calling [15]. Additional research has reported the negative stereotypes associated with eyeglasses wear across a variety of contexts [17,18]. Children who wear eyeglasses can be accused of “putting on airs” or trying to appear overly intellectual. Such teasing may spur a decline in psychological well-being [15]. It is therefore possible that such negative impacts may obscure or even completely outweigh mental health gains accrued by being able to see clearly and improve in school.

For this reason, we are interested in particular about how study intensity serves to mediate the impact of vision correction on mental health. Study intensity, as reflected in the number of hours spent studying outside of class, has been shown to predict perseverance and passion for long-term goals [19,20,21]. Studies have found that students who spend more time studying outside of class tend to be more disciplined and capable of self-control [19], and have better academic performance and higher attendance in class [14,22]. Such students may be more likely to ignore teasing or concerns of personal appearance because the benefit gained in learning is perceived to be more important. Conversely, students that study less intensively, who often have lower levels of academic performance, may be relatively more concerned with appearance and peer interactions, and thus be more vulnerable to teasing or other “side effects” of glasses wear. In this way, study intensity may moderate the impact of glasses on mental health as much as, or more than, other student characteristics such as family background or household wealth.

The overall goal of this study is to evaluate the effect of correcting refractive error on the mental health of students in schools in rural China. In particular, given China’s highly competitive schooling system, we are interested in exploring the differential impacts of vision correction on students with varying degrees of study intensity. To meet this goal, we have three specific objectives. First, we want to examine the average effect of correcting refractive error on the mental health condition of myopic students. Second, we seek to identify important heterogeneous effects of providing eyeglasses on mental health to determine whether vision correction affects the mental health of different types of children differently. Finally, we explore the mechanisms that might help to explain why correcting refractive error may have different effects on the mental health of students of different study intensity.

## 2. Sampling, Data Collection, and Analytical Approach

The setting for the study is the public school system in two of China’s predominantly rural northwestern provinces, Shaanxi and Gansu. Success in China’s competitive public school system is overwhelmingly dependent on performance on high-stakes tests [13], and only a fraction of students are able to move up through the school system and attend academic high school and college. Unfortunately, rural students often face a number of constraints, including poor access to quality health care, nutrition, and vision care. Further, rates of myopia are high among students in rural areas, approximately 20 to 30% in primary school, more than 50% in junior high, and as high as 90% upon completion of high school. As noted earlier, however, rates of eyeglasses wear remain very low among students.

We conducted a cluster-randomized controlled trial (hereafter, cluster-RCT) across 252 primary rural schools in China to study the impact of providing eyeglasses on the mental health of myopic students. In the trial, schools were randomly selected into the treatment group and the control group. Among 19,934 students in the 252 primary schools, 2851 myopic students who could potentially benefit from having eyeglasses were involved in the experiment. In the treatment group, fully subsidized eyeglasses were provided to eligible myopic students. In the control group, myopic students were given only eyeglasses prescriptions. Each myopic student was given a letter for their parents informing them of their child’s myopia status and prescription. No other action was taken. If the students in the control group opted to purchases glasses, they would also have to travel to the county seat and select an optical shop and purchase the eyeglasses.

### 2.1. Sampling

Sampling was conducted immediately prior to the implementation of the study during the 2012–2013 school year in one prefecture in Gansu Province and one prefecture in Shaanxi Province. By choosing two provinces that differ in terms of wealth and development, as measured by GDP per capita (Gansu is relatively poor, and Shaanxi is close to China’s overall average), we are able to increase the generalizability of our findings [23]. Gansu’s GDP per capita, 3919 USD, was ranked the second poorest among China’s 31 provincial administrative regions in 2013. Shaanxi’s GDP per capita, 6883 USD, was ranked 12th [24]. One prefecture in each province was selected, Tianshui in Gansu Province and Yulin in Shaanxi Province. In total, we collected data in 18 of the 19 counties in the two prefectures. One county was not included due to the small size of its population, the surveying of which would have substantially raised the cost of the survey. 

To choose the sample schools and students in these two prefectures, we followed a three-step protocol. First, we obtained a list of all rural primary schools in the two prefectures from local county education bureaus. Second, to eliminate the potential spillovers, we randomly selected only one school from each school district (or town) in the sample frame. Finally, within each school, we randomly selected one class in each of the fourth and fifth grades (likely age range was 9–12 years old). In total, 19,934 students from 252 rural primary schools were selected. 

### 2.2. Experimental Design

To ensure a balanced sample and to improve the power of the experimental design, an intervention assignment was stratified by location (county), school size, and eye examination results (i.e., the proportion of myopic students in each school) collected in the baseline survey. In total, this yielded a total of 45 strata, and our analysis takes this stratified randomization procedure into account [25]. Two-thirds of the schools were randomly assigned to one of two treatment groups and the other one-third to a control group. Among the two treatment groups, half of the schools were assigned to a free eyeglasses treatment group and the other half to a voucher treatment group. Because the intervention in both treatment groups was almost the same (at least in the context of this study, as both sets of students received the same vision care and were given access to the kind of eyeglasses), we combined the two treatment groups into one.

Once the randomized assignment was completed, the implementation team launched the intervention. In the treatment group, every student was screened. Students with poor vision were identified through a two-step eye examination protocol (described in detail in Section 2.3). A prescription for eyeglasses for each student with poor vision was produced by a professional optometrist. A letter that described this vision care program and a prescription for the student was sent to parents. In addition, information about vision care and the importance of wearing eyeglasses was given to students in the form of an in-class training program. Based on the prescription, a pair of free eyeglasses was produced for each myopic student. About four weeks after the baseline (October 2012) program optometrists dispensed free eyeglasses at schools for the free treatment group, and free eyeglasses were ready for pickup at a shop of a local optometrist for the voucher treatment group. There were not large differences in acceptance or the wearing of eyeglasses or in the impact on academic outcomes between the two treatment groups, which further supports the decision to combine the two treatment groups in this study. 

In the control group, only the letter and a prescription for the student were given to the parents. No additional training was provided to the students. The students and their families were blind to the cluster-RCT. 

### 2.3. Data Collection

We conducted a total of two waves of surveys: one at baseline and one at post-treatment. Each survey was administrated to all sample students in the 252 schools. The baseline survey was conducted in September 2012 at the beginning of fall semester. The post-treatment survey was conducted in June 2013 at the end of the spring semester. 

#### 2.3.1. Baseline Survey 

Baseline surveys were administered to all students in a classroom in two blocks. In the first block, examiners administered questionnaires to students in regard to age, gender, eyeglasses wear, awareness of refractive status, knowledge of vision care, and boarding status at school. The questionnaires also contained a place for an estimate of the number of hours that each student studied after class per day. In this study, we use this measure as a proxy for each student’s study intensity. Each student was asked to record how much time he or she spends studying outside of school, drawing from a list of options that included “0–0.5 h,” “0.5–2 h”, or “more than 2 h.” Students who recorded studying more than 2 h were considered to maintain high study intensity, whereas those who indicated studying for less than half an hour per day were considered students of low study intensity.

The first block of the survey also included a parent survey form in which parents reported their migration status, family asses, and education. We collected detailed information on the migration status of each student’s parents. Specifically, the questions asked whether each parent had been migrated out of the area for work during the past six months. Field observations and interviews with key informants suggest that for most rural laborers in the study area, if they are working and living away from home for at least six months, it is almost certain that they were actually away from home for most or all of the entire year [26,27]. As a way of cross checking, we asked the homeroom teacher to verify the information on the parental migration status of each student. In our overall sample, around 12.5% of children are left behind children (that is, both parents have out-migrated), just a bit lower than estimates of the average of representative rural areas in China (15.7%) [28]. 

The household survey also collected information on value of family assets that students would likely have difficulty answering. Caregivers were asked to fill out a checklist of thirteen household consumption assets: automobile, truck, motor bike, tractor, farming equipment, computer, internet, television, camera, washing machine, air conditioner, water heater, gas stove, refrigerator, kitchen ventilator, and flushable toilet. A value was attached to each asset (based on the National Household Income and Expenditure Survey, which is organized and published by the China National Bureau of Statistics) to produce a single metric of household asset holdings. Summing the value of all household consumption assets then produced our proxy variable for family wealth value [29]. We did so because recent studies suggest using household asset indicators for household wealth is more reliable than self-reported income [30].

In the second block, examiners administered a test that we used to assess the mental health condition of each student. In our study, we measured mental health with an instrument that researchers in China term the Mental Health Test (MHT). The MHT was developed by Prof. Bucheng Zhou of East China Normal University, who adapted it from the General Anxiety Test developed by Kiyoshi Suzuki in Japan [31]. The MHT is a variation of the Children’s Manifest Anxiety Scale, which is an internationally standardized test for the anxiety of children that has been widely used in the United States and other developed countries [32]. The MHT also has been widely applied in China [7,30]. The test has a reliability of 0.84–0.88 and a retest reliability of 0.78–0.86. Among the subcategories of the scale, we pay special attention to learning anxiety and physical anxiety, which are the two most common anxieties among rural students in China [30]. The use of eyeglasses would give myopic students a clear view of the blackboard and learning materials, thus potentially reducing their anxiety towards learning and physical health.

According to the MHT scale, learning anxiety refers to a student’s fear of examinations or excessive concerns about test scores. Such as, worried about passing exams successfully, feel unhappy when the test scores are not good, feel anxious when student cannot remember what he/she has learned during an examination, and worry about getting a poor score when taking an exam. Physical anxiety refers to a student’s excessive concerns about his/her body, such as: always worried about there is something wrong in his/her body, often think that other students are prettier or more handsome than him/her, find it difficult to sleep at night, and unwilling to take medicine.

During the analysis, we normalized the anxiety scores against the control group’s baseline distribution. Higher scores represent higher levels of anxiety.

At the same time as the baseline survey, a two-step eye examination was administered to all students in the randomly selected classes in all sample schools. In the first step, a team of two trained staff administered visual acuity (VA) screenings, using Early Treatment Diabetic Retinopathy Study eye charts. Students who failed the VA screening test (the cutoff is a VA of either eye of less than or equal to 6/12, or 20/40) were enrolled in a second vision test that was conducted at each school one to two days after the first test. The second vision test was conducted by a team of one optometrist, one nurse, and one assistant. It involved cycloplegic automated refraction with subjective refinement to determine prescriptions for children’s eligibility for eyeglasses (the cutoff for myopia is ≤−0.5 diopters [D]). The eye examination team was trained by Zhongshan Ophthalmic Center (ZOC) at Sun Yat-sen University. 

#### 2.3.2. Post-Treatment Survey 

In May 2013, approximately seven months after the eyeglasses were dispensed, a post-treatment survey was conducted. The post-treatment survey followed the same protocol as the baseline survey. As in the baseline survey, the examiners collected post-treatment MHT scores. As in the case of the baseline survey, we normalized scores, using the control group’s baseline distribution.

As we were conducting the post-treatment survey, we also conducted an unannounced spot check to collect information on the eyeglasses wear of myopic students. We also conducted an unannounced spot check to collect information on the eyeglasses wear of myopic students. A team of two examiners was sent to schools in advance of the rest of the survey team. Examiners were given a list of the students diagnosed with myopia in the baseline and recorded individual-level information on whether each student wears eyeglasses regularly when studying and outside of class. Eyeglasses wear is a binary variable based on whether students wear eyeglasses regularly.

Right after the spot check, a survey that is similar to the baseline one was administered to all students in a classroom. In addition, being aware that misperceptions about wearing eyeglasses might lead myopic students to be teased by their classmates, we asked each student whether myopic students were being teased in his or her class. Being teased is a binary variable. We also collect data on whether students consider wearing eyeglasses to be good looking. Specifically, we define eyeglasses are good looking as a binary variable taking the value of one if a student thinks wearing eyeglasses is good looking.

### 2.4. Summary Statistics, Balance, and Attrition

Given our sampling strategy, the results of our baseline survey are representative of students in poor rural areas in China. As seen in Table 1, students in our sample are, on average, 10.51 years old (or 10 years, 6 months), which suggests that students started primary school (Grade 1), on average, at 7 years old. In our overall sample, 52% are male, which is similar to the sex ratio across poor areas of rural China [12]. In our sample, only 8.7% of mothers and 13.3% of fathers completed more than 12 years of education. For adults of this age, the levels of education are also typical as across China, where only 11.3% of individuals aged 25–64 from rural areas finished upper secondary education [30]. Finally, in our overall sample, 12.5% of children are left-behind children (that is, both parents have out-migrated). This level is similar to (or slightly lower than) estimates of the share of children who are left behind in China (15.7%) [18].

To identify students with vision problems, optometrists who worked with the survey team conducted eye examinations with all students in the 252 sample schools. Of the total number of students in the overall sample (19,934), 2851 (14.3%) were myopic to a degree that they could benefit from having eyeglasses; these students were included in our analysis (Table 1).

Random assignment successfully created a sample balanced between the treatment and control groups. Using student individual characteristics, family characteristics, and student mental health scores, Table 1 shows that the groups were similar in terms of the measured characteristics. A joint significance test across all baseline characteristics also confirms that the study arms are balanced. We test this by regressing treatment status on all baseline characteristics reported in Table 1 and test that the coefficients on all characteristics were jointly zero. The p-value of this test is 0.2344 (treatment group vs. control group). The attrition rate of the sample between baseline and post-treatment was 3.9%. In the RCT literature, this attrition rate is considered low [33]. 

Table A1 indicates that there are no statistically significant differences in the rates of attrition between the treatment group and control group. Further, as seen in Table A2, baseline characteristics are still well balanced between the control and treatment groups in terms of non-missing observations. In a later analysis, we control for these variables in the regression.

### 2.5. Statistical Approach

We use ordinary least squares (OLS) regression analysis to estimate the treatment effect and heterogeneous impacts.

First, we compare the post-treatment standardized MHT scores and learning anxiety scores of students between the treatment and control schools, controlling for baseline standardized math test scores and a set of controls with strata effect and school effect.

The specification of the model is as follows:

(1) $$Y_{1ijp}=\beta_{0}+\beta_{1}Z_{ijp}+\beta_{2}Y_{0ijp}+X_{ijp}^{\prime }\gamma +Strata_{j}+School_{jp}+\epsilon_{ijp}$$

where $Y_{1ijp}$ is the post-treatment standardized MHT scores, learning anxiety scores, and physical anxiety scores for student *i* at school p in stratum *j*, treatment dummy $Z_{ijp}$ takes the value of 1 if students were provided with free eyeglasses, and $Y_{0ijp}$ is the standardized MHT, learning anxiety, and physical anxiety scores in the baseline. $X_{ijp}$ is a vector of other baseline characteristics (described in Table 1). 

Given that providing free eyeglasses might have a heterogeneous impact on students with different study intensity at baseline, we use the following model to estimate the heterogeneous treatment effect:

(2) $$Y_{1ijp}=\beta_{0}+\beta_{1}Z_{ijp}+\beta_{2}Z_{ijp}\times 1\{D_{0ijp}=2\}+\beta_{3}Z_{ijp}\times 1\{D_{0ijp}=3\}+\;\theta Y_{0ijp}+X_{ijp}^{\prime }\gamma +Strata_{j}+School_{jp}+\epsilon_{ijp}$$

where we add interaction terms of the treatment dummy and the indicator functions of the subgroup of study intensity at baseline $D_{0ijp}$, which takes three values: 1, 2 and 3. The parameter $\beta_{1}$ is a measure of the impact of providing free eyeglasses on the subgroup of students who study at a low level of intensity (0 to 0.5 h of studying per day after class); $\beta_{1}+\beta_{2}$ measures the impact on the subgroup of students who study at a medium level of intensity (0.5 to 2 h of studying per day after class); and $\beta_{1}+\beta_{3}$ measures the impact on the subgroup of students who study at a high level of intensity (greater than 2 h of studying per day after class).

## 3. Results

### 3.1. Impact of Providing Free Eyeglasses/Wearing Eyeglasses on Mental Health

The impact of the eyeglasses promotion program on mental health is presented in Table 2. Estimation from ordinary least squares regression analysis using model (1) show that the treatment has small and insignificant for Learning Anxiety and MHT. There is, however, a significant reduction in Physical Anxiety (Table 2, column 2). 

Although there is no impact of either providing free eyeglasses or wearing eyeglasses on mental health on average, it is worth considering whether there might be a heterogeneous treatment effect between different subgroups. It could be the case that there are heterogeneous treatment effects in subgroups with opposite signs that offset each other. 

The results indicate that the treatment affects students of varying degrees of study intensity differently (Table 3). Treatment students who study at a low intensity level experience a 0.17 SD rise in Learning Anxiety, significant at the 5% level. Conversely, treatment students who study at a high intensity level experience a 0.25 SD decline in Learning Anxiety, significant at the 10% level; a 0.22 SD decline in Physical Anxiety, significant at the 10% level; and a 0.26 SD improvement in MHT score, significant at the 5% level. Echoing this trend, treatment students who study at a moderate degree of intensity experience a 0.13 decline in MHT score, significant at the 5% level. 

In summary, the intervention of providing free eyeglasses, on average, does not improve the mental health condition of students. Even when we consider the effect of wearing eyeglasses on mental health (average treatment effect on the students), the estimated coefficient is still statistically insignificant, except for a small decline in Physical Anxiety. When we estimate the heterogeneous impacts as varying by study intensity, interestingly, we find the treatment worsens learning anxiety for students with low study intensity, whereas it improves the mental health condition for students with higher study intensity.

### 3.2. Mechanisms that Drive the Impact of Heterogeneity

The results presented in Table 4 concerns possible mechanisms for the heterogeneous impacts presented above. The results from regressions without controlling for baseline characteristics show a similar finding, see Table A4 and Table A5. The analysis concerns secondary outcomes—Eyeglasses Wear, Change in Study Hours, Math Score, Being Teased, and Finding Eyeglasses Good Looking—among students of varying degrees of study intensity. 

All students, regardless of their level of study intensity, experience a rise in wear of eyeglasses due to the intervention. Students who study at a high level of intensity experience an 18 percent rise in eyeglasses wear, significant at the 1% level, while moderate- and low-intensity students experience somewhat lower rate of wearing eyeglasses compare to their high study intensity peers (Table 4, column 1).

The analysis of the Being Teased outcome may shed light on why low intensity students experience a rise in Learning Anxiety, while their high intensity peers enjoy a decline in Learning Anxiety (as reported in Table 3). Students who study at a low intensity level experience a 13 percent rise in being teased from their peers due to the intervention, significant at the 1% level (Table 4, column 4). Moderate-intensity students see a similar, if more moderate, 11 percent rise in being teased, significant at the 1% level. High-intensity students experience no change in the amount that they are teased. A possible mechanism for this finding is that increasingly teased children internalize the notion that they are being perceived as more studious on account of wearing glasses, and therefore feel slightly more pressure to perform in school. No similar scenario unfolds for high intensity students, perhaps because they are already used to performing well in school. We examined the difference in baseline math test scores between different study intensity groups. Students who spend less time studying (low study intensity) at baseline were indeed the students who had worst test scores, they were 0.34 standard deviation behind the moderate study intensity students and 0.27 standard behind the high study intensity students, see Table A3. This interpretation is buttressed by the fact that high intensity students report a rise in believing that eyeglasses are good looking (Table 4, column 5)—after all, they are not being teased for wearing them. 

One seemingly contradictory finding from Table 4 is that students who get teased more as a consequence of wearing glasses still wear the eyeglasses. However, we see from the analysis that the vision correction intervention raised test scores by 0.13 SD among low study intensity myopic students, significant at the 1% level (Table 4, column 3). This reveals a possible explanatory factor for the apparently contradictory finding. The gains achieved in test score outcomes may be perceived as more important than the costs associated with increased teasing. Therefore, the children persist in wearing their glasses.

Finally, no students see any change in their hours of study due to the intervention. This appears to suggest that changes in the mental health outcomes among students in all three study intensity groups were not caused by any change in study time. 

## 4. Conclusions

In this study, we use a cluster-RCT to measure the effect of an eyeglasses promotion program in 252 Chinese rural primary schools on the mental health condition of myopic students. On average, the treatment has small and insignificant for Learning Anxiety and MHT, and a small but significant reduction in Physical Anxiety. 

These average treatment effects mask important heterogenous effects, however. Heterogeneous analysis on the amount of time that students spend in studying after school reveals that the program reduces both Learning Anxiety and Physical Anxiety among students who study with a high degree of intensity. Children who study less intensively are shown to experience an increase in Learning Anxiety. Increased teasing of less studious students following their acceptance of eyeglasses may explain their rise in anxiety. 

The strengths of the current study include its large sample size and randomized design. The study of the mental health impacts of myopia correction also fills a gap in the literature concerning refractive error care among children, particularly as it relates to their study intensity. Although the sample area was large, caution must be used in extrapolating the results across all rural areas of China or beyond. 

The study can inform policy on the provision of vision care in schools in important ways. It is clear from earlier analyses that the timely correction of myopia boosts academic outcomes for students [1]. In-school screenings and the subsidization of eyeglasses are also important drivers of eyeglasses acceptance and wear. Wear rates among students who need eyeglasses remain well below 100% in most contexts. To boost wear rates and minimize negative spillovers on mental health for some students, care should be taken in the classroom to eliminate teasing of students who are newly wearing eyeglasses, and encouragement should be offered to students of all types to recognize their eyeglasses as the valuable learning asset that they are.

## Figures and Tables

**Table 1:** Baseline Characteristics across Experimental Groups.

Variable	Entire Sample	Control Group	Treatment Group	Difference	*p*-Value
Student level					
Age (years)	10.507	10.547	10.487	−0.0597	0.4272
	(1.104)	(1.109)	(1.102)		
Male, 1 = yes	0.485	0.496	0.48	−0.0165	0.4097
	(0.5)	(0.5)	(0.5)		
Refractive error, D	−2.232	−2.277	−2.211	0.0659	0.2628
	(1.245)	(1.271)	(1.232)		
Wear eyeglasses at baseline, 1 = yes	0.154	0.145	0.159	0.0134	0.4808
	(0.361)	(0.353)	(0.365)		
Math score	0.239	0.244	0.236	−0.0079	0.9003
	(0.989)	(1.006)	(0.982)		
MHT score	−0.034	−0.037	−0.033	0.0041	0.4025
	(1.042)	(1.048)	(1.04)		
Learning anxiety score	−0.021	−0.052	−0.006	0.0461	0.948
	(1.014)	(0.999)	(1.022)		
Physical anxiety score	−0.045	−0.048	−0.043	0.0047	0.9365
	(0.999)	(1.028)	(0.985)		
Family level					
One or both parents with > 12 years education, 1 = yes	0.206	0.203	0.207	0.0039	0.8466
	(0.404)	(0.403)	(0.405)		
Family asset value, thousand RMB	32.06	30.763	32.679	1.9161	0.5567
	(35.394)	(34.215)	(35.936)		
Number of observations	2851	929	1922	2851	

Note: Standard deviations in parentheses.

**Table 2:** Impact of Providing Fully Subsidized Eyeglasses on Mental Health.

Dependent Variable	Post-Treatment		
	Learning Anxiety	Physical Anxiety	MHT
Treatment, 1 = yes	−0.0308	−0.0763 ^†^	−0.0781
	(0.0528)	(0.0449)	(0.0492)
Variable controlled			
Baseline learning anxiety	Yes	No	No
Baseline physical anxiety	No	Yes	No
Baseline MHT	No	Yes	Yes
Student, family characteristics	Yes	Yes	Yes
Control mean	−0.1170	-0.1310	−0.1840
Number of observations	2557	2557	2557

Note: Standard deviations in parentheses. All coefficients are estimated with mixed-effect linear regression clustering at strata and school levels. † *p* < 0.10. MHT: Mental Health Test.

**Table 3:** Impact of Subsidized Eyeglasses on Mental Health by After-class Study Time.

Dependent Variable	Post-Treatment		
	Learning Anxiety	Physical Anxiety	MHT
Treatment, 1 = yes	0.1740 *	−0.0379	0.0940
	(0.0857)	(0.0740)	(0.0815)
Baseline study hours 0–0.5 h (as comparison)			
Baseline study hours 0.5–2 h, 1 = yes	0.1603 ^†^	0.0245	0.1314
	(0.0829)	(0.0722)	(0.0799)
Baseline study hours >2 h, 1 = yes	0.3558 **	0.1656	0.2324 ^†^
	(0.1265)	(0.1102)	(0.1219)
Treatment x (Baseline study hours 0.5–2 h)	−0.2696 **	−0.0292	−0.2258 *
	(0.1001)	(0.0870)	(0.0963)
Treatment x (Baseline study hours >2 h)	−0.4192 **	−0.1789	−0.3511 *
	(0.1524)	(0.1325)	(0.1466)
Variable controlled			
Baseline learning anxiety	Yes	No	No
Baseline physical anxiety	No	Yes	No
Baseline MHT	No	No	Yes
Student, family characteristics	Yes	Yes	Yes
Treatment effect for 0–0.5 h	0.1740 *	−0.0379	0.0940
*p*-value for 0–0.5 h	0.0420	0.6080	0.2490
Treatment effect for 0.5–2 h	−0.0960	−0.0670	−0.1320 *
*p*-value for 0.5–2 h	0.1440	0.2310	0.0320
Treatment effect for >2 h	−0.2450 ^†^	−0.2170 ^†^	−0.2570 *
*p*-value for >2 h	0.0620	0.0570	0.0410
*N*	2557	2557	2557
Control mean	−0.1170	−0.1310	−0.1840

Note: Cluster-robust standard errors in parentheses. All coefficients are estimated with mixed-effect linear regression clustering at strata and school levels. ^†^ *p* < 0.10, * *p* < 0.05, ** *p* < 0.01.

**Table 4:** Impact of Providing Fully Subsidized Eyeglasses on Secondary Outcomes by Daily After-class Study Time.

Dependent Variables	Post-Treatment				
	(1) Wear Eyeglasses	(2) Change Study Hours	(3) Math Score	(4) Being Teased	(5) Eyeglasses Good-Looking
Treatment, 1 = yes	0.1073 **	0.0006	0.1248 ^†^	0.1261 **	0.0022
	(0.0378)	(0.0368)	(0.0652)	(0.0335)	(0.0290)
Baseline Study hours 0–0.5 h	(as comparison)				
Baseline Study hours 0.5–2 h, 1 = yes	0.0265	−0.2431 **	0.1505 **	0.0021	0.0004
	(0.0324)	(0.0363)	(0.0584)	(0.0312)	(0.0254)
Baseline Study hours >2 h, 1 = yes	0.0380	0.2257 **	0.1823 *	0.0422	−0.0118
	(0.0499)	(0.0554)	(0.0899)	(0.0479)	(0.0392)
Treatment * (Study hours 0.5–2 h)	0.0069	0.0062	−0.0419	−0.0168	0.0068
	(0.0393)	(0.0439)	(0.0709)	(0.0379)	(0.0309)
Treatment * (Study hours >2 h)	0.0690	−0.0057	−0.0976	−0.0545	0.0707
	(0.0603)	(0.0667)	(0.1085)	(0.0578)	(0.0473)
Variable Controlled:					
Baseline MHT, Learning Anxiety & Physical Anxiety	Yes	Yes	Yes	Yes	Yes
Student, Family Characteristics	Yes	Yes	Yes	Yes	Yes
Treatment effect for 0–0.5 h	0.1070 *	0.0010	0.1250 ^†^	0.1260 **	0.0020
*p*-value for 0–0.5 h	0.0050	0.9870	0.0560	0.0000	0.9400
Treatment effect for 0.5–2 h	0.1140 **	0.0070	0.0830	0.1090 **	0.0090
*p*-value for 0.5–2 h	0.0000	0.8060	0.1140	0.0000	0.7040
Treatment effect for >2 h	0.1760 **	−0.0050	0.0270	0.0720	0.0730 ^†^
*p*-value for >2 h	0.0010	0.9280	0.7770	0.1570	0.0860
*N*	2538	2631	2537	2536	2534
Control Mean	0.2690	0.4750	0.3280	0.1390	0.1150

Note: Cluster-robust standard errors in parentheses. All coefficients are estimated with mixed-effect linear regression clustering at strata and school levels. ^†^ *p* < 0.10, * *p* < 0.05, ** *p* < 0.01.

## Appendix A

**Table A1:** Attrition check.

Dependent Variable	Attrition, 1 = Yes
Treatment, 1 = yes	0.014
	(0.009)
Control mean	2851
Number of observations	0.030
Overall attrition rate	0.039

Note. All coefficients are estimated with mixed-effect linear regression clustering at strata and school levels.

**Table A2:** Balance check of baseline characteristics of the remaining sample across experimental groups.

Variable	Control Group	Treatment Group	Difference	*p*-Value
Student level				
Age (years)	10.549	10.481	−0.0676	0.3673
	(1.1)	(1.084)		
Male, 1 = yes	0.502	0.481	−0.0207	0.3107
	(0.5)	(0.5)		
Refractive error, D	−2.285	−2.215	0.0699	0.2459
	(1.274)	(1.236)		
Wear eyeglasses at baseline, 1 = yes	0.142	0.16	0.0183	0.3356
	(0.349)	(0.367)		
Baseline math score	0.25	0.235	−0.0155	0.806
	(1.002)	(0.981)		
Baseline MHT score	−0.057	−0.03	0.0274	0.6688
	(1.044)	(1.038)		
Baseline learning anxiety score	−0.068	−0.004	0.0645	0.2529
	(0.999)	(1.022)		
Baseline physical anxiety score	−0.061	−0.04	0.0208	0.7293
	(1.026)	(0.981)		
Family level				
One or both parents with > 12 years education, 1 = yes	0.202	0.203	0.0009	0.9662
	(0.402)	(0.402)		
Family asset value	30.422	32.884	2.4622	0.4482
	(33.728)	(36.082)		
Number of observations	901	1840	2741	

Note. Standard deviation in parentheses.

**Table A3:** Summary of student baseline math scores, by after-class study hours.

	Low Intensity (0–0.5 h)	Moderate Intensity (0.5–2 h)	High Intensity (>2 h)	Mild vs. Low	High vs. Low	High vs. Moderate
	(1)	(2)	(3)	(2)–(1)	(3)–(1)	(3)–(2)
Math Score	0.01	0.35	0.28	0.34 **	0.27 **	−0.071
	(0.974)	(0.969)	(1.028)	(0.042)	0.0732	(0.0639)

Note. Means with standard deviations reported in brackets; Cluster-robust standard errors adjusted for clustering at the school level in parentheses. ** *p* < 0.01.

**Table A4:** Impact of Providing Fully Subsidized Eyeglasses on Mental Health by Daily After-class Study Time (without controls).

Dependent Variable	Post-Treatment		
	Learning Anxiety	Physical Anxiety	MHT
Treatment, 1 = yes	0.1606 ^†^	0.0100	0.1279
	(0.0923)	(0.0813)	(0.0937)
Baseline study hours 0–0.5 h	(as comparison)		
Baseline study hours 0.5–2 h, 1 = yes	0.1342	0.0458	0.1591 ^†^
	(0.0874)	(0.0784)	(0.0897)
Baseline study hours >2 h, 1 = yes	0.3186 *	0.2482 *	0.3274 *
	(0.1327)	(0.1189)	(0.1362)
Treatment x (Baseline study hours 0.5–2 h)	−0.2066 ^†^	−0.0334	−0.2087 ^†^
	(0.1062)	(0.0953)	(0.1091)
Treatment x (Baseline study hours >2 h)	−0.3058 ^†^	−0.1802	−0.3472 *
	(0.1608)	(0.1440)	(0.1649)
Variable controlled			
Baseline learning anxiety	No	No	No
Baseline physical anxiety	No	No	No
Baseline MHT	No	No	No
Student, family characteristics	No	No	No
Treatment effect for 0–0.5 h	0.1610 ^†^	0.0100	0.1280
*p*-value for 0–0.5 h	0.0820	0.9020	0.1720
Treatment effect for 0.5–2 hrs	−0.0460	−0.0230	−0.0810
*p*-value for 0.5–2 h	0.5170	0.7040	0.2580
Treatment effect for >2 h	−0.1450	−0.1700	−0.2190
*p*-value for >2 h	0.2970	0.1690	0.1230
*N*	2765	2765	2765
Control mean	−0.1310	−0.1440	−0.1990

Note: Cluster-robust standard errors in parentheses. All coefficients are estimated with mixed-effect linear regression clustering at strata and school levels. ^†^ *p* < 0.10, * *p* < 0.05.

**Table A5:** Impact of Providing Fully Subsidized Eyeglasses on Secondary Outcomes by Daily After-class Study Time (without controls).

Dependent Variables	Post-Treatment				
	(1)	(2)	(3)	(5)	(6)
	Wear Eyeglasses	Change Study Hours	Math Score	Being Teased	Eyeglasses Good-Looking
Treatment, 1 = yes	0.1105 **	−0.0060	0.1110	0.1173 **	0.0173
	(0.0400)	(0.0359)	(0.0802)	(0.0329)	(0.0280)
Baseline Study hours 0–0.5 h	(as comparison)				
Baseline Study hours 0.5–2 h, 1 = yes	0.0361	−0.2529 **	0.3745 **	−0.0122	0.0182
	(0.0335)	(0.0347)	(0.0702)	(0.0301)	(0.0246)
Baseline Study hours >2 h, 1 = yes	0.0315	0.2150 **	0.2306 *	0.0534	−0.0024
	(0.0513)	(0.0525)	(0.1074)	(0.0459)	(0.0377)
Treatment * (Baseline Study hours 0.5−2 h)	−0.0050	0.0099	−0.0936	−0.0077	−0.0108
	(0.0410)	(0.0423)	(0.0859)	(0.0368)	(0.0301)
Treatment * (Baseline Study hours >2 h)	0.0807	−0.0052	0.0222	−0.0530	0.0465
	(0.0624)	(0.0636)	(0.1305)	(0.0557)	(0.0457)
Variable Controlled:					
Student, Family Characteristics	No	No	No	No	No
Treatment effect for 0−0.5 h	0.1110 **	−0.0060	0.1110	0.1170 **	0.0170
*p*-value for 0−0.5 h	0.006	0.8670	0.1670	0.0000	0.5350
Treatment effect for 0.5−2 h	0.1060 **	0.0040	0.0170	0.1100 **	0.0070
*p*-value for 0.5−2 h	0.001	0.8870	0.7880	0.0000	0.7700
Treatment effect for >2 h	0.1910 **	−0.0110	0.1330	0.0640	0.0640
*p*-value for >2 h	0.001	0.8370	0.2530	0.1880	0.1170
*N*	2740	2850	2739	2738	2739
Control Mean	0.2640	0.4840	0.3200	0.1400	0.1170

Note: Cluster-robust standard errors in parentheses. All coefficients are estimated with mixed-effect linear regression clustering at strata and school levels. * *p* < 0.05, ** *p* < 0.01.
